# Acute gastrointestinal and genitourinary toxicity of image-guided intensity modulated radiation therapy for prostate cancer using a daily water-filled endorectal balloon

**DOI:** 10.1186/1748-717X-7-76

**Published:** 2012-05-23

**Authors:** Curtiland Deville, Stefan Both, Viet Bui, Wei-Ting Hwang, Kay-See Tan, Mattia Schaer, Zelig Tochner, Neha Vapiwala

**Affiliations:** 1 Department of Radiation Oncology, Pennsylvania, USA; 2 Department of Biostatistics & Epidemiology, University of Pennsylvania, Philadelphia, Pennsylvania, USA; 3 University of Pennsylvania, Perelman School of Medicine, Department of Radiation Oncology, 3400 Civic Center Boulevard, TRC 2W, Philadelphia, PA 19104, USA

**Keywords:** Rectal balloon, IMRT, Toxicity, Prostate cancer

## Abstract

**Background:**

Our purpose was to report acute gastrointestinal (GI) and genitourinary (GU) toxicity rates for prostate cancer patients undergoing image-guided intensity modulated radiation therapy (IG-IMRT) with a daily endorectal water-filled balloon (ERB_H2O_), and assess associations with planning parameters and pretreatment clinical characteristics.

**Methods:**

The first 100 patients undergoing prostate and proximal seminal vesicle IG-IMRT with indexed-lumen 100 cc ERB_H2O_ to 79.2 Gy in 1.8 Gy fractions at our institution from 12/2008- 12/2010 were assessed. Pretreatment characteristics, organ-at-risk dose volume histograms, and maximum GU and GI toxicities (CTCAE 3.0) were evaluated. Logistic regression models evaluated univariate association between toxicities and dosimetric parameters, and uni- and multivariate association between toxicities and pretreatment characteristics.

**Results:**

Mean age was 68 (range 51–88). Thirty-two, 49, and 19 patients were low, intermediate, and high-risk, respectively; 40 received concurrent androgen deprivation. No grade 3 or greater toxicities were recorded. Maximum GI toxicity was grade 0, 1, and 2 in 69%, 23%, and 8%, respectively. Infield (defined as 1 cm above/below the CTV) rectal mean/median doses, D75, V30, and V40 and hemorrhoid history were associated with grade 2 GI toxicity (Ps < 0.05). Maximum acute GU toxicity was grade 0, 1, and 2 for 17%, 41%, and 42% of patients, respectively. Infield bladder V20 (P = 0.03) and pretreatment International Prostate Symptom Scale (IPSS) (P = 0.003) were associated with grade 2 GU toxicity.

**Conclusion:**

Prostate IG-IMRT using a daily ERB_H2O_ shows low rates of acute GI toxicity compared to previous reports of air-filled ERB IMRT when using stringent infield rectum constraints and comparable GU toxicities.

## Introduction

Dose-escalated radiotherapy (RT) as radical treatment for clinically localized prostate cancer is well-established. Increasing dose is limited by surrounding normal tissue toxicity, and improved tumor control may come at the expense of increased toxicity, particularly rectal. Modern RTechniques including three-dimensional conformal (3DCRT), intensity modulated (IMRT), proton therapy, and daily image guidance (IG) have allowed for more conformal prostate dose distributions with improved normal tissue sparing. Additionally, a daily endorectal balloon (ERB) has been implemented for 1) prostate immobilization to reduce planning target volume (PTV) margins [1], [2] and 2) rectal wall sparing [3], [4].

An air-filled endorectal balloon (ERB_air_) has been used in photon therapy for the potential anterior rectal wall (ARW) surface-sparing effect. Dose perturbation near the air-tissue interface occurs due to electronic disequilibrium, leading to lower doses in tissues adjacent to the air cavity. Monte Carlo (MC) calculations of parallel-opposed photon beams have shown dose reductions up to 21%, 15%, and 11%, at the air-rectum interface and 1 and 2 mm depths respectively [5], [6], while a 15% dose reduction at the air-tissue interface was observed using multiple-beam IMRT [7]. Despite these apparent advantages, this technique also poses a dosimetric challenge, because the air introduces significant heterogeneity, Since almost three-quarters of prostate cancer foci are present in the peripheral zone [8] in close rectal proximity this has led to concern of potential target under-dosing. When comparing the Eclipse treatment planning system to MC simulation using a four-field box technique and no heterogeneity corrections, Song, et al. found that the treatment planning system predicted higher mean doses, and they concluded that a potential underdosage of 3.4% mean dose for the posterior beam near the peripheral zone of the prostate was quantified [9]. Although improvement in dose calculation in heterogeneous media was made within the Eclipse TPS when transitioning from the single pencil beam algorithm (SPB) to the analytical anisotropic algorithm, further improvement may be still necessary especially for dose calculated in the heterogeneous interface area [10].

A daily water-filled endorectal balloon (ERB_H2O_) has been routinely employed in proton therapy for dose distribution optimization [11]. Use of an ERB_H2O_ during IMRT, rather than air-filled balloon, reduces dosimetric concern of this dose heterogeneity and potential diminished target coverage posteriorly. In order to maintain the favorable rectal toxicity profile, stringent rectal planning constraints were applied. This study analyzes our preliminary institutional experience using this approach, reporting the rates of acute genitourinary (GU) and gastrointestinal (GI) toxicity and assessing for toxicity associations with dose-volume histogram (DVH) parameters as well as patient characteristics. This is the first report to our knowledge on reporting the acute toxicity of IG-IMRT using an ERB_H2O_.

## Methods and materials

We retrospectively analyzed the first 100 patients undergoing prostate/proximal seminal vesicle IG-IMRT to 79.2 Gy in 1.8 Gy fractions at our institution between 12/2008 – 12/2010.

### Clinical evaluation

Patients had histologically-confirmed, clinically localized prostate adenocarcinoma evaluated by complete history, physical examination including digital rectal examination, bone scintigraphy, and computed tomography (CT) of the abdomen and pelvis and/or pelvic magnetic resonance imaging (MRI) ± endorectal coil. Pelvic lymph nodes were not included in the irradiated target volume. Concurrent androgen deprivation therapy (ADT), consisting of LHRH analog administration prior to RT initiation, was at the physician’s discretion and offered primarily to patients with adverse risk features (PSA >10, cT3a/T3b, and/or Gleason ≥7).

### *Radiotherapy planning and delivery*

Prior to RT, three electromagnetic transponders (Calypso®, Calypso Medical Technologies, Inc, Seattle, WA) or static fiducial markers (Visicoil™, IBA Dosimetry, Bartlett, TN) were implanted into the prostate (right base, left base, and apex) under transrectal ultrasound guidance. Planning CT was performed at minimum 4 days after implantation on a CT simulator (Siemens USA, New York, NY). Pre-CT bowel and bladder preparation included dietary guidelines, regular use of anti-gas tablets, two self-administered enemas to ensure an empty rectum, and at least 500 mL of water 20 to 30 minutes prior to simulation to achieve a full bladder. (Daily enemas were not required during treatment, however, instructions to arrive with an empty rectum and full bladder were reinforced). Patients were immobilized supine with Knee-Lok and Foot-Lok cushions (CIVCO, Orange City, IA), and an indexed lumen, 9 cm ERB (RadiaDyne, LLC, Houston, TX) was inserted into the rectum and filled with 100 mL of water. A 1.5-mm slice scan was acquired at prostate level and isocenter placed in the geometric center of the markers. The prostate and proximal two-thirds of the seminal vesicles were outlined by the physician and identified as the clinical target volume (CTV). The PTV was generously defined as a 1-cm elliptical expansion of the CTV in all directions, except 0.6 cm posteriorly. Minimum CTV and PTV coverage was D98 >98% and D95 >95%, respectively. Organs at risk (OARs) evaluated in this study were 1) bladder: contoured from base to dome, 2) bladder wall: defined as a 3 mm internal rind of the bladder surface), 3) rectum: from anus (at ischial tuberosity level) to rectosigmoid flexure, and 4) anterior rectal wall (ARW): defined as 3 mm rind of the anterior rectal circumference. “Infield” OARs, defined as 1 cm above and below CTV, were also created for each structure. Predefined institutional volumetric target dose constraints included: bladder V65 < 20%, V50 < 45%, and V45 < 50% and infield rectum V60 < 20%, V45 < 40%, and V40 < 50%. When necessary, acceptable deviations were consistent with dose level 3 of RTOG 9406 and considered ‘minor’ if within 5% of institutional goals. OAR DVHs were reviewed. IMRT plans were generated using 7–9 coplanar fields (at least 4 of which were posterior oblique fields traveling through the rectum) with 6 and/or 15 MV photon beams. Patients were treated with Varian linear accelerators (Varian 2300IX; Varian Medical Systems, Palo Alto, CA). Setup accuracy was verified for patients with daily kV-kV portal images to ensure prostate fiducial marker alignment, or in the case of patients with Calypso beacons, transponder CT coordinates and isocenter information were imported from Eclipse (Varian Medical Systems), and after appropriate patient setup adjustments, the system was used in continuous tracking mode throughout treatment delivery.

### *Clinical assessment*

Patients were monitored weekly during treatment and seen within 1–3 months after RT completion. At each visit, a history was obtained with emphasis on treatment-related morbidity. Toxicities were scored according to The Common Terminology Criteria for Adverse Events version 3.0 [12] and International Prostatic Symptom Scale (IPSS) [13]. Maximum grade toxicity rates were compared.

### *Statistical analysis*

Baseline patient characteristics – age, race/ethnicity, PSA, clinical tumor stage, Gleason score, risk group, concurrent ADT use, type of prostate fiducial marker, prostate volume, medical co-morbidity (hypertension (HTN), hyperlipidemia, or diabetes mellitus (DM)), pre-RT urinary signs/symptoms, and pretreatment IPSS – were reported.

Using logistic regression analysis, statistical associations were investigated between grade ≥2 GU toxicity and (1) bladder dosimetric parameters and (2) pretreatment demographics, including age, PSA, pathologic tumor stage, Gleason score, concurrent ADT, HTN, hyperlipidemia, DM, IPSS, and pre-RT GU past medical history (PMH) defined as an antecedent history of significant urinary sign or symptoms, including benign prostatic hyperplasia, prior trans-urethral resection of the prostate, bladder outlet obstruction, and/or lower urinary tract symptoms. Similarly, associations were investigated separately between grade ≥ 2 and (1) rectal dosimetric parameters and (2) the aforementioned pretreatment demographics, excluding IPSS and pre-RT GU PMH. Pretreatment demographics with significant univariate toxicity associations (p values of <0.10) were considered in a multivariate logistic regression model. Given relatively low numbers of Grade ≥2 toxicities, to preserve robust estimation, no more than two variables were included in the final model. Although multiple statistical tests were performed, due to the preliminary nature of the analysis, a p value of <0.05 was considered statistically significant. Statistical analyses were conducted using Stata version 11 software (StataCorp, College Station, TX).

## Results

All patients tolerated the ERB throughout the entire treatment course. Pretreatment demographics are listed in Table 1. The majority of patients had clinical stage T1c, Gleason 6 or 7 disease, median PSA 7.6, mean age 68, mean prostatic volume 45 cc, and mean IPSS 8.

**Table 1:** Characteristics for the study sample

**N**	**100**
Mean Age ± SD (range)	67.5 ±8.3 (51 – 88)
Race	%
White	37
Black	50
Asian	4
Other, Unknown, Hispanic	9
Median PSA (range)	7.6 (1–40.9)
T stage	%
T1c	90
T2a	7
T3a	2
T3b	1
Gleason Score	%
6	32
7	54
8	7
9	6
Risk group	%
Low risk	32
Intermediate risk	49
High risk	19
Co-morbid conditions	%
GU PMH	34
HTN	72
DM	27
Hyperlipidemia	47
Hemorrhoids	11
ADT	40
Calypso®	38

Abbreviations: N = number of patients; SD = standard deviation; IPSS = International Prostate Symptom Score; BAS = Bowel Assessment Score; PSA = prostate specific antigen; ADT = androgen deprivation therapy; GU = genitourinary; PMH = past medical history; HTN = hypertension; DM = diabetes mellitus.

### *GI toxicity*

Toxicities are summarized in Table 2; no grade ≥3 toxicities were observed. Maximum GI toxicity was grade 0, 1, and 2 in 69%, 23%, and 8%, respectively. One patient reported grade 2 enteritis using oral anti-diarrheal medication. Seven patients reported grade 2 rectal proctitis or bleeding, 3 (43%) of which reported a history of hemorrhoids prior to treatment initiation, while only 2/14 (14%) with grade 1 and 6/79 (7.6%) with grade 0 lower GI tract toxicity reported similar history. On univariate analysis, only hemorrhoid history was significantly associated (P = 0.025) with GI grade 2 toxicity.

**Table 2:** Acute Maximum GU and GI Toxicities with IMRT using a water-filled endorectal balloon

	**GU**	**GI**
**Toxicity Grade**	**%**	**%**
0	17	69
1	41	23
2	42	8
3	0	0
4	0	0

Abbreviations: GU = genitourinary; GI = gastrointestinal; IMRT = intensity modulated radiation therapy.

### *GU toxicity*

Maximum GU toxicity was grade 2 (42%), grade 1 (41%), and grade 0 (17%). Grade 2 GU toxicity consisted of frequency (24%), urgency (13%), dysuria (6%), incontinence (4%), obstruction (2%), and retention (2%) with some patients reporting multiple grade 2 toxicities. The majority of acute grade 2 GU events involved initiation or increase in alpha-blocker or less frequently, anti-spasmodic. The mean pretreatment IPSS was 8 ± 7 (0–34), and the mean subjective IPSS score was 2 ± 1 (0–5). The mean post-treatment IPSS was 10 ± 6 (1–24), and the mean subjective IPSS score was 2 ± 1 (0–5). The mean absolute change in pre- and post-treatment IPSS was 2 ± 5 (−7 - 16). The mean percentage change in pre- and post-treatment IPSS was 6% ± 15% (−20% - 46%). Thus, the IPSS increased on average 2 points from pre- to immediate post-treatment recording, while the subjective IPSS score on average was unchanged, confirming the tolerability and mild bother of such treatment.

Thirty-four percent of patients had an antecedent GU PMH, however this was not associated with the occurrence of grade ≥2 GU toxicity. On univariate analysis, concurrent ADT (P = 0.08) and IPSS values pre-RT (P = 0.003), post-RT (P = 0.018), absolute increase (P = 0.013), and percentage increase (P = 0.013) – were all associated with acute grade ≥2 GU toxicity. Only pre-treatment IPSS remained significant (P = 0.003) on multivariate analysis.

### *Dosimetry and dosimetric-toxicity associations*

IMRT plans provided excellent coverage, with all plans achieving target coverage goals. No hot spots were located within the OAR or OAR-target volume interfaces. Six patients did not have all bladder constraints met, 4 of whom had single deviations <5%. Twelve patients did not have all infield rectum constraints met, 9 of whom had a single deviation <2%. Figure 1 shows the mean DVHs for the reported OARs. Tables 3 and 4 show the mean ± standard deviation of the DVH parameters assessed for the reported OARs, as well as the univariate association with grade ≥2 GI and GU toxicity, respectively. On logistic regression analyses, only infield bladder V_20_ was associated with the occurrence of grade ≥2 toxicity (P = 0.033), while the infield bladder wall V_20_ was marginally associated (P = 0.061). Grade 2 GI toxicity was associated with infield rectal mean/median doses, D_75_, V_30_, and V_40_ (Ps < 0.05), and did not associate with rectum, ARW and infield ARW parameters.

**Table 3:** Rectum RTOG, Anterior Rectal Wall RTOG, Rectum Partial, Anterior Rectal Wall Partial DVH Analysis and Univariate Associations with Grade ≥ 2 GI Toxicities

**Grade ≥ 2 Combined GI/rectal**	**Rectum**		**Infield Rectum**		**Anterior Rectal Wall**		**Infield Anterior Rectal Wall**	
	**Mean ± SD**	**P value**	**Mean ± SD**	**P value**	**Mean ± SD**	**P value**	**Mean ± SD**	**P value**
Volume (cm^3^)	167.1 ± 31.4	0.57	131.5 ± 25.3	0.59	26.9 ± 5.7	0.63	20.7 ± 4.6	0.54
D_min_ (Gy)	4.2 ± 3.7	0.64	12.3 ± 4.6	0.08	5.8 ± 5.8	0.87	20.6 ± 7.3	0.17
D_max_ (Gy)	81.8 ± 1.7	0.44	81.8 ± 1.7	0.44	81.8 ± 1.7	0.40	81.8 ± 1.7	0.40
D_mean_ (Gy)	36.0 ± 5.1	0.28	42.1 ± 4.1	0.04	54.2 ± 7.1	0.82	64.6 ± 3.1	0.48
D_median_ (Gy)	31.7 ± 5.9	0.09	35.9 ± 5.2	0.03	60.5 ± 8.7	0.91	70.9 ± 4.4	0.73
V_10_ (%)	87.4 ± 10.4	0.99	99.3 ± 2.1	0.64	88.6 ± 10.1	0.81	99.9 ± 0.3	NA
V_20_ (%)	77.1 ± 14.5	0.46	91.5 ± 11.6	0.21	84.7 ±11.2	0.92	99.6 ± 1.3	0.39
V_30_ (%)	57.1 ± 14.5	0.08	69.2 ± 15.3	0.02	80.8 ± 11.5	0.79	97.6 ± 3.4	0.30
V_40_ (%)	35.4 ± 7.6	0.13	43.4 ± 8.4	0.046	72.8 ± 11.5	0.76	89.6 ± 6.0	0.35
V_50_ (%)	24.2 ± 4.5	0.53	29.9 ± 4.8	0.24	62.1 ± 10.4	0.95	77.5 ± 7.2	0.90
V_60_ (%)	17.0 ± 3.0	0.85	21.3 ± 3.3	0.48	51.8 ± 9.2	0.91	65.6 ± 7.2	0.96
V_65_ (%)	14.0 ± 2.5	0.87	17.5 ± 2.9	0.51	46.6 ± 8.5	0.98	59.3 ± 7.1	0.81
V_70_ (%)	10.9 ± 2.2	0.86	13.7 ± 2.7	0.54	40.8 ± 8.1	0.95	52.2 ± 7.5	0.71
V_75_ (%)	7.2 ± 2.2	0.74	9.1 ± 2.7	0.55	32.2 ± 8.4	0.75	41.4 ± 9.4	0.58
V_79.2_ (%)	2.4 ± 2.1	0.49	3.0 ± 2.5	0.37	12.7 ± 9.8	0.42	16.4 ± 12.2	0.31

Abbreviations: DVH = dose volume histogram; D_min_ = minimum dose; D_median_ = median dose; D_mean_ = mean dose; D_max_ = maximum dose; V_10_, V_20_, V_30_, V_40_, V_50,_ V_60_, V_65_, V_70_, V_75,_ V_79.2_ = volume receiving 10 Gy, 20 Gy, 30 Gy, 40 Gy, 50 Gy, 60 Gy, 65 Gy, 70 Gy, 75 Gy, 79.2 Gy, respectively.

Note: The results are presented as mean ± standard deviation. The results listed as NA were not estimated due to the limited number of variations in the tested parameter.

**Table 4:** Bladder, Bladder Wall, Proximal Bladder, and Proximal Bladder Wall DVH Analysis and Univariate Associations with Grade ≥ 2 GU Toxicities

	**Bladder**		**In-field Bladder**		**Bladder Wall**		**In-field Bladder Wall**	
	**Mean ± SD**	**P value**	**Mean ± SD**	**P value**	**Mean ± SD**	**P value**	**Mean ± SD**	**P value**
Volume (cm^3^)	246 ± 167	0.31	101.5 ± 42.9	0.34	55.2 ± 24.6	0.44	25.9 ± 8.1	0.65
D_min_ (Gy)	3.6 ± 4.7	0.92	13.0 ± 5.9	0.55	3.6 ± 4.7	0.94	12.5 ± 6.6	0.21
D_max_ (Gy)	82.5 ± 1.6	0.26	82.5 ± 1.6	0.26	82.4 ± 1.6	0.24	82.4 ± 1.6	0.24
D_mean_ (Gy)	31.0 ± 11.2	0.64	50.4 ± 5.8	0.16	33.2 ± 10.6	0.72	54.9 ± 5.7	0.23
D_median_ (Gy)	23.1 ± 15.2	0.62	48.6 ± 8.1	0.16	23.4 ± 16.2	0.60	56.0 ± 9.9	0.20
V_10_ (%)	66.6 ± 25.1	0.66	99.2 ± 3.2	0.11	64.6 ± 22.8	0.61	99.0 ± 2.9	0.14
V_20_ (%)	57.1 ± 24.1	0.55	94.6 ± 7.6	0.03	56.2 ± 20.9	0.66	94.5 ± 6.5	0.06
V_30_ (%)	46.4 ± 20.4	0.65	81.3 ± 11.1	0.20	47.1 ± 17.5	0.84	82.6 ± 9.5	0.37
V_40_ (%)	35.4 ± 15.0	0.65	64.6 ± 11.9	0.48	38.7 ± 13.6	0.86	69.7 ± 10.5	0.57
V_50_ (%)	25.4 ± 10.1	0.40	48.0 ± 11.4	0.27	30.5 ± 10.4	0.61	56.1 ± 10.9	0.30
V_60_ (%)	17.9 ± 7.3	0.46	34.6 ± 10.0	0.32	24.2 ± 8.5	0.63	45.4 ± 10.6	0.34
V_65_ (%)	15.1 ± 6.3	0.41	29.4 ± 9.2	0.26	21.9 ± 7.8	0.59	41.3 ± 10.1	0.30
V_70_ (%)	12.4 ± 5.4	0.38	24.6 ± 8.3	0.23	19.7 ± 7.1	0.56	37.5 ± 9.6	0.29
V_75_ (%)	9.5 ± 4.4	0.32	18.9 ± 7.4	0.22	16.9 ± 6.4	0.45	32.6 ± 9.3	0.24
V_79.2_ (%)	4.7 ± 3.0	0.45	9.9 ± 6.5	0.48	10.2 ± 5.3	0.52	20.6 ± 10.4	0.45

Abbreviations: DVH = dose volume histogram; D_min_ = minimum dose; D_median_ = median dose; D_mean_ = mean dose; D_max_ = maximum dose; V_10_, V_20_, V_30_, V_40_, V_50,_ V_60_, V_65_, V_70_, V_75,_ V_79.2_ = volume receiving 10 Gy, 20 Gy, 30 Gy, 40 Gy, 50 Gy, 60 Gy, 65 Gy, 70 Gy, 75 Gy, 79.2 Gy, respectively.

Note: The results are presented as mean ± standard deviation.

## Discussion

This study is the first to our knowledge to report the acute toxicity of prostate cancer patients treated with IG-IMRT using an ERB_H2O_. Grade ≥2 GI and GU toxicity rates of 8% and 42%, respectively, compare favorably with patients treated with IMRT using an ERB_air_ for which there is only single institution data reporting rates of acute GI and GU toxicity of 18% and 35%, respectively, in 396 prostate cancer patients treated from 1997–2001 with mean dose 77 Gy (specifically 70 Gy in 2 Gy daily fractions prescribed to the 85% isodose line) IMRT using 100 cc ERB_air_[14]. The toxicity rates for our cohort are also comparable to the more extensively reported acute GI and GU toxicity rates for non-ERB prostate IMRT including from our own institution at 13% and 50%, respectively [15]. These comparative rates for acute GI toxicity are summarized in Table 5. While PTV margins were the same in our ERB and non-ERB cohorts, in these initial 100 patients treated with a water-filled balloon and IG-IMRT, more stringent constraints were implemented for the bladder and rectum in this cohort and may explain the more favorable toxicity profile. Following our intrafraction prostate motion analysis [2], we have subsequently reduced our PTV margins to 5 mm, except 4 mm posteriorly. 

**Table 5:** Comparative Rates of Acute Maximum RTOG Grade GI Toxicity for Prostate IMRT with no Endorectal Balloon, an Air-filled Endorectal Balloon, and a Water-filled Endorectal Balloon

	**non-ERB**^**15**^	**ERB**_**air**_^**14**^	**ERB**_**H2O**_
**Toxicity Grade**	**%**	**%**	**%**
0	63	68	69
1	23	14	23
2	13	18	8
3	0	0	0
4	0	0	0

Abbreviations: GU = genitourinary; GI = gastrointestinal; IMRT = intensity modulated radiation therapy; ERB = endorectal balloon; H2O – water.

Only hemorrhoid history was significantly associated on univariate analysis with grade 2 GI toxicity. Pre-existing hemorrhoid history has varied in significance in prior ERB studies. In one study, patients with pre-existing hemorrhoids did not have an increased risk of complaints [16], while in another it was considered a contraindication for the use of ERBs, as application led to grade 3 anal irritation in a patient with hemorrhoids [17]. In the Baylor report, patients with pre-existing anorectal disease had a higher risk of developing acute anorectal toxicity; 43% with pre-existing history of anorectal disease vs. 16% without reported acute grade 2 GI toxicity [14].

DVH-toxicity analysis revealed no association for the rectum when anatomically contoured per RTOG guidelines. Contrastingly, infield rectum mean/median doses, D_75_, V_30_, and V_40_ were associated with combined upper and lower GI grade 2 toxicity (Ps <0.05). In their initial acute toxicity report of the first 100 patients treated with IMRT utilizing an ERB_air_, the Baylor group found no relationship between acute rectal toxicity and rectal mean dose or rectal V65, V70, and V75 [18]. In that study, rectal delineation was not specified, and intermediate dose level parameters were not assessed. Dosimetric data assessing specifically acute GI toxicity associations and rectal parameters are also limited in the non-ERB IMRT setting. As the QUANTEC report recently summarized, most of the mature published clinical data on dose-related rectal toxicity come from non-ERB 3DCRT and focus on either cumulative or late toxicity, concluding that the high-dose regions are most predictive [19]. Still, particularly when considering ERB use, our findings are consistent with their conclusion that reduced rectal volumes exposed to intermediate dose ranges by IMRT may become more important in assessing associations with toxicity.

The GU toxicity profile of prostate IMRT using an ERB_H2O_ is not expected to vary from ERB_air_ and non-ERB prostate IMRT, as the dose distribution to the bladder is not significantly altered with ERB use. As noted above, GU toxicity rates remain consistent overall. On univariate and multivariate analysis, pre-treatment IPSS remained significant, confirming its continued relevance in predicting likelihood of acute grade ≥2 GU toxicity with ERB use. There were no associations with bladder or bladder wall DVH parameters. Similarly, the Baylor group found no relationship between acute GU toxicity and bladder DVH parameters [14], corroborating reports that bladder DVH parameters generally do not consistently correlate with acute or late GU toxicities. Still, in the follow-up setting, there have been reports noting similar volumetric associations with late GU toxicity, demonstrating that the bladder wall V30 > 30 cc and V82 > 7 cc [20] and the fractional bladder volume receiving more than 14 to 27 Gy [21] were clinically useful predictors. In this analysis, infield bladder V_20_ was associated with the occurrence of grade ≥2 toxicity, while the infield bladder wall V_20_ was marginally associated, suggesting that further investigation of infield bladder DVH parameters may be relevant in the analysis of acute toxicity.

This is preliminary report is limited by its retrospective, nonrandomized nature. We await increased numbers and follow-up for further analysis including biochemical control, correlates with acute and eventually late toxicity, and comparison to non-ERB patients. PTV margins have subsequently reduced to 5 mm except 4 mm posteriorly based on our motion analysis papers, which may allow for further reductions in volumes of bladder and rectum irradiated potentially affecting the toxicity profile. Additional limitations are the tools used for toxicity assessment: CTCAE grading and IPSS. More comprehensive systems, such as the complete Expanded Prostate cancer Index Composite (EPIC) [22] - since implemented in our clinic - may assess subtler, but still clinically relevant morbidity, particularly assessing the bowel function and bother domains. Finally, as noted in the statistical analysis section, due to the preliminary and exploratory nature of the dosimetry-toxicity association analyses, although multiple statistical tests were performed, a p value of <0.05 was still considered statistically significant, however, it is therefore possible that p-values < 0.05 were based on chance. Despite these limitations, we felt that a preliminary report was merited, as the acute toxicity profile of IG-IMRT using an ERB_H2O_ has not yet been reported in the literature.

## Conclusion

Prostate IG-IMRT using a daily ERB_H2O_ shows low rates of acute GI toxicity compared to previous reports of ERB_air_ IMRT when using strict infield rectum constraints and comparable GU toxicities. Comprehensive dosimetric analysis of this cohort revealed that intermediate dose-level infield rectal (mean/median doses, D75, V30, and V40) and low dose in-field bladder (V20) parameters were associated with acute toxicity, as well as, hemorrhoid history and pretreatment IPSS.

## Competing interests

The author(s) declare that they have no competing interests.

## Authors’ contributions

CD, NV, SB, and ZT conceived of the study, and participated in its design and coordination and helped to draft the manuscript. VB and MS participated in its design, data collection, and statistical analyses. WH and KT participated in its design, carried out the statistical analyses, and helped to draft the manuscript. All authors read and approved the final manuscript.
